# Adherence to antiretroviral therapy among cisgender gay, bisexual and other men who have sex with men in Brazil: Evaluating the role of HIV-related stigma dimensions

**DOI:** 10.1371/journal.pone.0308443

**Published:** 2024-08-08

**Authors:** Victor C. Matos, Thiago S. Torres, Paula M. Luz

**Affiliations:** 1 Escola Nacional de Saúde Pública Sergio Arouca, Fundação Oswaldo Cruz, Rio de Janeiro, RJ, Brazil; 2 Instituto Nacional de Infectologia Evandro Chagas, Fundação Oswaldo Cruz, Rio de Janeiro, RJ, Brazil; University of Technology Sydney, AUSTRALIA

## Abstract

**Background:**

In Brazil, ~35% of people living with HIV (PLHIV) have poor adherence to antiretroviral therapy (ART). HIV-related stigma is associated with worst continuum of care outcomes, however evidence from Brazil is scarce. We explored pathways between HIV-related stigma dimensions and ART adherence among Brazilian cisgender gay, bisexual and other men who have sex with men (MSM) living with HIV.

**Methods:**

A sample of MSM ≥18 years was recruited online between February/March 2020 through advertisements on *Hornet*, a location-based dating app. Validated scales were used to assess ART adherence and HIV-related stigma. Indirect and direct pathways between HIV-related stigma dimensions and ART adherence were estimated using structural equation models while considering socio-demographic and substance use related variables. Models were estimated using mean- and variance-adjusted weighted least squares, and goodness of fit indices were calculated.

**Findings:**

Among 1,719 MSM living with HIV who reported starting ART, 70% were adherent. There was evidence of indirect effects of concerns about public attitudes (standardized coefficient (SC) = -0.095, 95% confidence interval (95%CI) = -0.172 - -0.017) and personalized HIV-stigma (SC = -0.022, 95%CI = -0.043 - -0.001) on ART adherence mediated through negative self-image. Personalized HIV stigma and concerns about public attitudes were both positively associated with negative self-image (SC = 0.129, 95%CI = 0.066–0.193; SC = 0.549, 95%CI = 0.494–0.603), and concerns about public attitudes was associated with HIV disclosure concerns (SC = 0.522, 95%CI = 0.463–0.581). However, the direct paths from personalized HIV stigma and concerns about public attitudes to ART adherence were not significant.

**Interpretation:**

Our research underscores the critical need for multifaceted interventions to eliminate HIV-related stigma at both individual and societal levels. At the individual level, psychotherapeutic interventions to improve self-image might helpful. Additionally, public policy should aim to dismantle structural stigma with awareness campaigns on various media channels, integration of anti-stigma curriculum into schools, and training for professionals.

## Introduction

Antiretroviral therapy (ART) has changed the course of the HIV epidemic and currently grants people living with HIV (PLHIV) to have a similar life expectancy to the general population in high-income countries [1] and in Latin America [2]. Additionally, adherence to ART hinders HIV transmission [3], making adherence central to ending the HIV epidemic. Nonetheless, suboptimal adherence is a reality in many chronic diseases [4], including HIV infection [4, 5], where it may lead to virologic failure and further the development of drug-resistant viral strains [6, 7]. Therefore, not only does ART nonadherence pose a threat to individual health, but it also constitutes a menace to public health by increasing community HIV viral load, HIV transmission risk, and the possibility of development of drug-resistant viral strains [8].

For antiretrovirals used during the first two to three decades of treatment, the threshold for optimal ART adherence stood at a minimum of 95%, owing to the achievement of virologic suppression at this level [9]. However, there has been significant improvement in pharmacokinetic profiles of newer antiretroviral medications, which can promote virologic suppression with lower levels of adherence, 85–89% [9] and 80–90% [10–12]. Moreover, the development of single-tablet daily regimens effectively addresses high pill burden, which is a recurrent barrier to medication adherence, ultimately facilitating ART adherence [13].

Nevertheless, despite these advancements in antiretrovirals’ efficacy, tolerability and posology, individuals still face numerous obstacles to achieving and sustaining optimal adherence in both high-income and middle or low-income countries [5]. In Brazil, a meta-analysis, which included studies using different adherence measures published between 2005 and 2016, estimated that the proportion of PLHIV with optimal ART adherence was 64% [14]. More recently, considerable attention has been given to HIV-related stigma, a psychosocial and structural factor that hinders adherence [15]. Similar to individuals living with other chronic health conditions, like Tuberculosis [16] and Hansen’s disease [17], PLHIV face societal stigmatization [18], which may lead to a loss of self-esteem, anxiety, negative self-evaluation and hesitation or even withdrawal from social interactions [19]. Moreover, HIV-related stigma has been shown to act as a barrier to engagement in HIV prevention, such as HIV testing, protected sex, and pre-exposure prophylaxis (PrEP) use [15, 18, 20].

The concept of HIV-related stigma is rooted on Goffman’s seminal work on stigma [21], in which stigma arises from a societal understanding of a given trait as a “deeply discrediting attribute”, reducing the lives of the bearers of such trait to a “tainted, discounted one”. Hence, living with HIV is the central attribute for the HIV-related stigma [22]. In light of its association with other stigmatized behaviors, such as same-sex sexual behavior, multiple sexual partnerships and injection drug use, HIV-related stigma is viewed as a notably complex phenomenon and theorized to impact PLHIV in four different forms (also referred to as dimensions) [22, 23]. Personalized HIV stigma or enacted stigma refers to experiences of discrimination, prejudice or stereotyping that a person living with HIV has already *lived*. Concerns about public attitudes or perceived stigma refers to concerns that a person living with HIV has about stigmatizing attitudes from others around him, her or them. HIV disclosure concerns or anticipated stigma refers to a person living with HIV’s distress over expected discriminatory, prejudicial, or stereotypical actions once others know his or her status. Lastly, negative self-image or internalized stigma involves the endorsement by someone living with HIV of negative societal labels and perceptions of PLHIV [22, 24].

There is a large body of literature pointing to a negative association between experiencing HIV-related stigma and ART adherence [22, 25, 26]. Notwithstanding, there is substantial heterogeneity in the assessment of HIV-related stigma including different quantitative and qualitative instruments and mixed approaches [26]. Theoretical and empirical studies suggest HIV-related stigma is a multidimensional construct, and mostly understood as a four-dimensional phenomenon as described previously. Nonetheless, most studies to date have evaluated the association between one particular HIV-related stigma dimension and ART adherence [26], although recent work has included more dimensions [24, 25, 27–29].

Studies found that negative self-image was a mediator in the pathway from personalized HIV stigma and concerns about public attitudes to ART adherence [24, 25]. Others have studied the mediating role from disclosure concerns to ART adherence of stress experience and alcohol use [30], of medication concerns [31], and of medication support and ART self-efficacy [32]. However, these studies did not take into account all four dimensions of HIV-related stigma, mostly analyzing the association between one or two dimensions. Such approach might suffer from measurement error, as it does not take into consideration the embedded correlation among all dimensions of HIV-related stigma [28]. Moreover, there is substantial evidence of a negative association of alcohol [33, 34] and substance use [35] with ART adherence. Nevertheless, few studies have attempted to test whether these behavior components act as mediators of the effect of HIV-related stigma on ART adherence.

To deepen the understanding of mediating roles, in this study we tested a conceptual model describing the association between two HIV-related stigma dimensions (personalized HIV stigma and concerns about public attitudes) and ART adherence among cisgender gay, bisexual and other men who have sex with men (MSM) in Brazil. We evaluated the mediating role of two other HIV-related stigma dimensions (negative self-image and HIV disclosure concerns), as well as of use of alcohol and other substances.

## Methods

### Study design

A cross-sectional convenience sample of Brazilian *Hornet* users who answered an online survey between 20 February and 29 March 2020. *Hornet* is a location-based dating app mostly used by MSM. Roughly one million app users were invited twice to complete the survey through direct message inbox, wherein no compensation was offered as per Brazilian research regulations. The questionnaire was administered via Alchemer® (https://www.alchemer.com/) and consisted of 118 questions, including five attention questions designed to identify careless participation [36]. Some questions were displayed to the user conditional on previous answers based on a branching logic. Respondents were able to change and review any of their answers and always had the possibility to choose a non-response option. Usability and technical functionality of the survey in different platforms were assessed before launching the study. Details of study design were described elsewhere [37].

### Study population

The study population comprised adult (≥18 years-old) *Hornet* users living in Brazil at the time of participation in the survey. Participants who 1) did not provide informed consent, 2) were younger than 18 years-old, 3) were not currently residing in Brazil, 4) identified themselves as cisgender women, 5) incorrectly answered any of the five attention questions, or 6) did not complete the questionnaire were excluded. When duplicated responses per internet protocol (I.P.) address were identified, only the first occurrence was retained in the study. For this analysis, we included only cisgender gay, bisexual and other men who have sex with men who reported to be living with HIV and to have initiated ART.

### Outcome of interest: Adherence to antiretroviral therapy

ART adherence was measured via the WebAd-Q instrument, which consists of three questions inquiring on the number of ART doses that were missed or incorrectly taken with the following response options: “yes”, “no” or “I don’t remember”. This measure was developed through interviews and focus groups with PLHIV in Brazil and was validated in the Brazilian context [38]. Participants were deemed adherent if they answered “no” to all three questions, meaning that “yes” and “I don’t remember” responses were assumed as non-adherence indicators as per instrument’s design. Complementarily, adherence to ART was also assessed with the question “Please mark below the value that corresponds to how much of your antiretroviral medication you took in the past 30 days” to be answered on a virtual slider varying from 0 to 100%; individuals who marked 100% were considered adherent [39, 40].

### Explanatory variables

#### Demographic

Demographic information included age, education, income and race. Education (completed) was inquired using standard levels as per Brazil’s system and then categorized into high school degree or less, undergraduate education, and graduate education or higher. Household income was assessed according to the average minimum wage in Brazil in 2020 (BR$1,039 which is equivalent to $214 USD using the average conversion rate of March/2020) and varied from zero (no income) to more than ten times the minimum wage (>$2,140 USD). Race was measured using the Brazilian Institute of Geography and Statistics’ (Instituto Brasileiro de Geografia e Estatística, IBGE) classification system [41], namely: White which includes people who identify as White or of European descent; Pardo which includes people who identify as mixed race or multiracial, with a combination of White, Black, or Indigenous ancestry; Black which includes people who identify as Black or of African descent; Indigenous which includes people who identify as Indigenous, belonging to one or more of the various Indigenous ethnic groups in Brazil; or Asian which includes people who identify as Asian or of Asian descent, such as Japanese, Chinese, Korean, and others. Given the limited number of Indigenous and Asian participants, and consistent with prior Brazilian research [37], these groups were combined with Pardo and White participants, respectively.

#### Alcohol and substance use

Alcohol use in the year prior to study participation was assessed by AUDIT-C, which comprises the first three questions of the Alcohol Use Disorders Identification Test (AUDIT) [42]. For each question, there are five response options valued from 0 to 4 points. The score for AUDIT-C is defined by adding up each question’s answer value, thus ranging between 0 and 12. For men, a score of 4 or more is considered an indicator of hazardous drinking [43]. Substance use in the six months prior to study participation was assessed for the following substances: cocaine, crack, cannabis, methamphetamine, and inhalants (other than poppers).

#### HIV-related stigma

The experience of HIV-related stigma was evaluated with the Short HIV Stigma scale, which consists of a reduced 12-item form of the original 40-item scale created by Berger et al [44, 45]. This instrument was translated to Brazilian Portuguese and validated in the Brazilian context [46]. Four different dimensions of HIV-related stigma were assessed with the scale (personalized HIV stigma, concerns about public attitudes, negative self-image and HIV disclosure concerns), each with three items, with responses provided on a 4-point Likert-scale ranging from strongly disagree (1) to strongly agree (4). Scoring is calculated by summing the three items’ scores for each dimension, thus each dimension’s score ranges from 3 to 12. Higher scores indicate a higher degree of stigma.

### Latent variables

ART adherence, the four dimensions of HIV-related stigma, alcohol use, substance use, and socioeconomic status were all modelled as latent variables. In order to minimize measurement error and avoid empirical underidentification, latent variables should be derived from the shared variance of at least three observed variables [47]. For the four dimensions of HIV-related stigma, and alcohol use, the items of their respective instruments were used to define the latent variables. For ART adherence, in addition to the three items of the WebAd-Q instrument, the dichotomized slider response was included to define the latent variable. Substance use was defined from five indicators, one for each substance: cocaine, crack, cannabis, methamphetamine and inhalants. Lastly, socioeconomic status’ latent variable was defined from three measured variables: education, income, and race.

### Theoretical model

**Fig 1:**
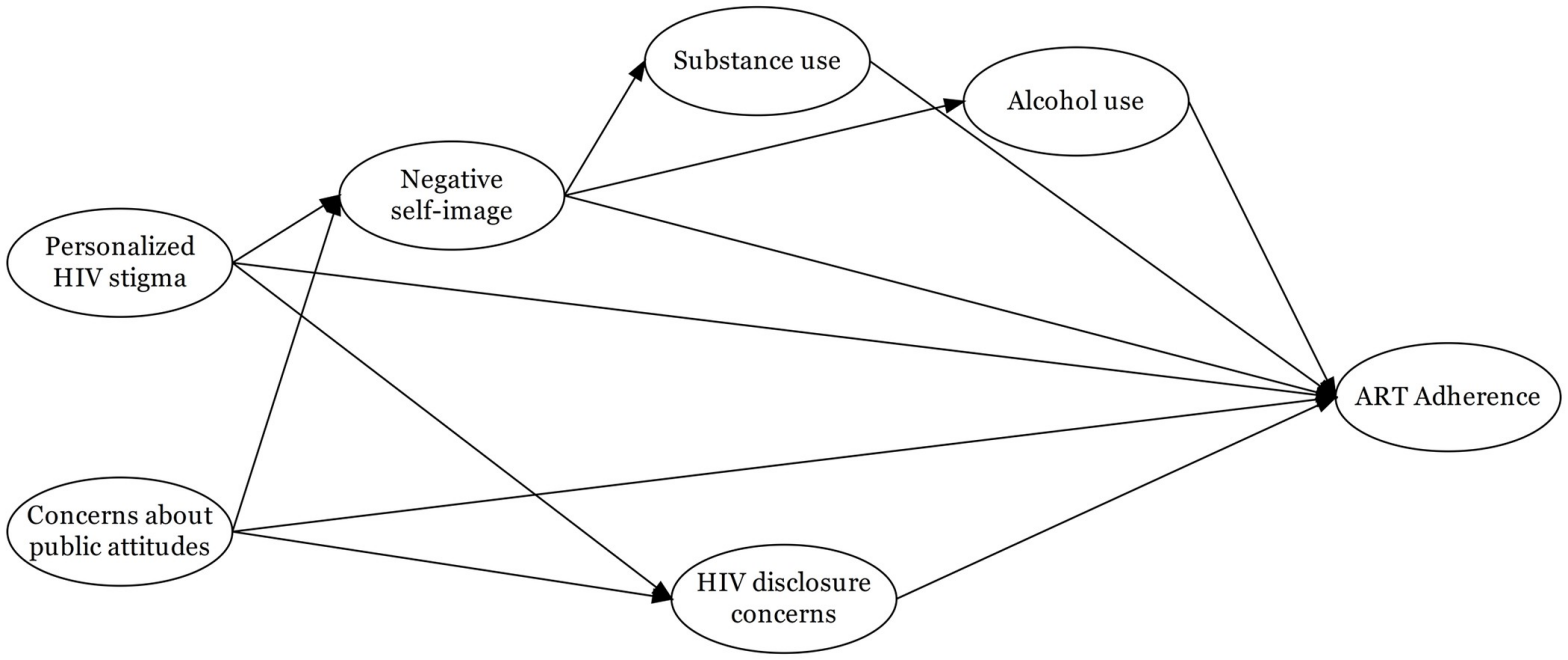
Proposed theoretical model to analyze the association between HIV-related stigma and adherence to therapy.

Earnshaw and Chaudoir’s Health Stigma Framework (HSF) presents personalized HIV stigma, negative self-image, and HIV disclosure concerns as relevant dimensions of how PLHIV experience and cope with HIV-related stigma [48]. This original conceptual model has been expanded to include concerns about public attitudes regarding PLHIV [24], and some HIV-related stigma dimensions have been understood as possible mediators of the relationship between other HIV-related stigma dimensions and ART adherence [49]. In light of the foregoing, we hypothesized that personal experiences of stigmatizing situations (personalized HIV stigma) and concerns about public attitudes regarding PLHIV play a role in the individual’s ART adherence directly and through other HIV-related dimensions and behaviors. One possible link from personalized HIV stigma and concerns about public attitudes to ART adherence is through the acceptance and endorsement of negative stereotypes attributed to PLHIV (negative self-image). There is also evidence showing that negative self-image is associated with alcohol use disorders and substance use, which in turn influences ART adherence [50–52]. Another link might occur via HIV disclosure concerns, where an individual’s effort to keep his HIV status secret from others limits his ability to engage in HIV care. Socioeconomic status (SES) and age were assumed as possible confounders of all associations drawn in the theoretical model shown in Fig 1.

### Statistical analysis

Descriptive statistics were calculated for all variables: for categorical variables, absolute numbers, and percentages; for continuous variables: mean, standard deviation (SD), median, interquartile range (IQR), minimum (min) and maximum (max). We used structural equation modeling (SEM) to evaluate the appropriateness of our measurement model (our measured latent variables) and to test whether our theoretical model was supported by the data. The direct and indirect effects of HIV-related stigma dimensions on ART adherence were assessed with SEM using weighted least squares adjusted for mean and variance (WLSMV). Standardized coefficients (SCs) were used to evaluate these effects, and they represent the change (increase) in standard deviation (SD) of the dependent variable given a SD increase in the independent variable, holding all other variables constant [47]. WSLMV estimator is robust to non-normal data and encompasses categorical variables. Missing data was present for 45 participants (22 had missing information for schooling and 37 had missing information for race/skin color) and was handled during the estimation process using pairwise deletion. According to our theoretical model, negative self-image and HIV disclosure concerns, alcohol and substance use were mediators of the association between personalized HIV stigma and concerns about public attitudes and the outcome. Negative self-image and HIV disclosure concerns were allowed to covary. Similar to other studies [24, 25, 28], SES and age were included as confounders in all paths. Model fit was assessed using root mean square error of approximation (RMSEA), comparative fit index (CFI), Tucker-Lewis index (TLI) and standardized root mean square residual (SRMR). Model fitting was deemed acceptable if CFI, TLI ≥ 0.95, RMSEA ≤ 0.06 and SRMR ≤ 0.08 [53]. Data preparation was performed in R version 4.2.3 and estimation in Mplus version 8.8.

### Ethics statement

This study received approval from the human subjects ethics committee at Instituto Nacional de Infectologia Evandro Chagas of Fundação Oswaldo Cruz (#CAAE 01777918.0.0000.5262). All study participants provided electronic informed consent before survey initiation. No personally identifiable information was collected, except for I.P. address.

## Results

**Fig 2:**
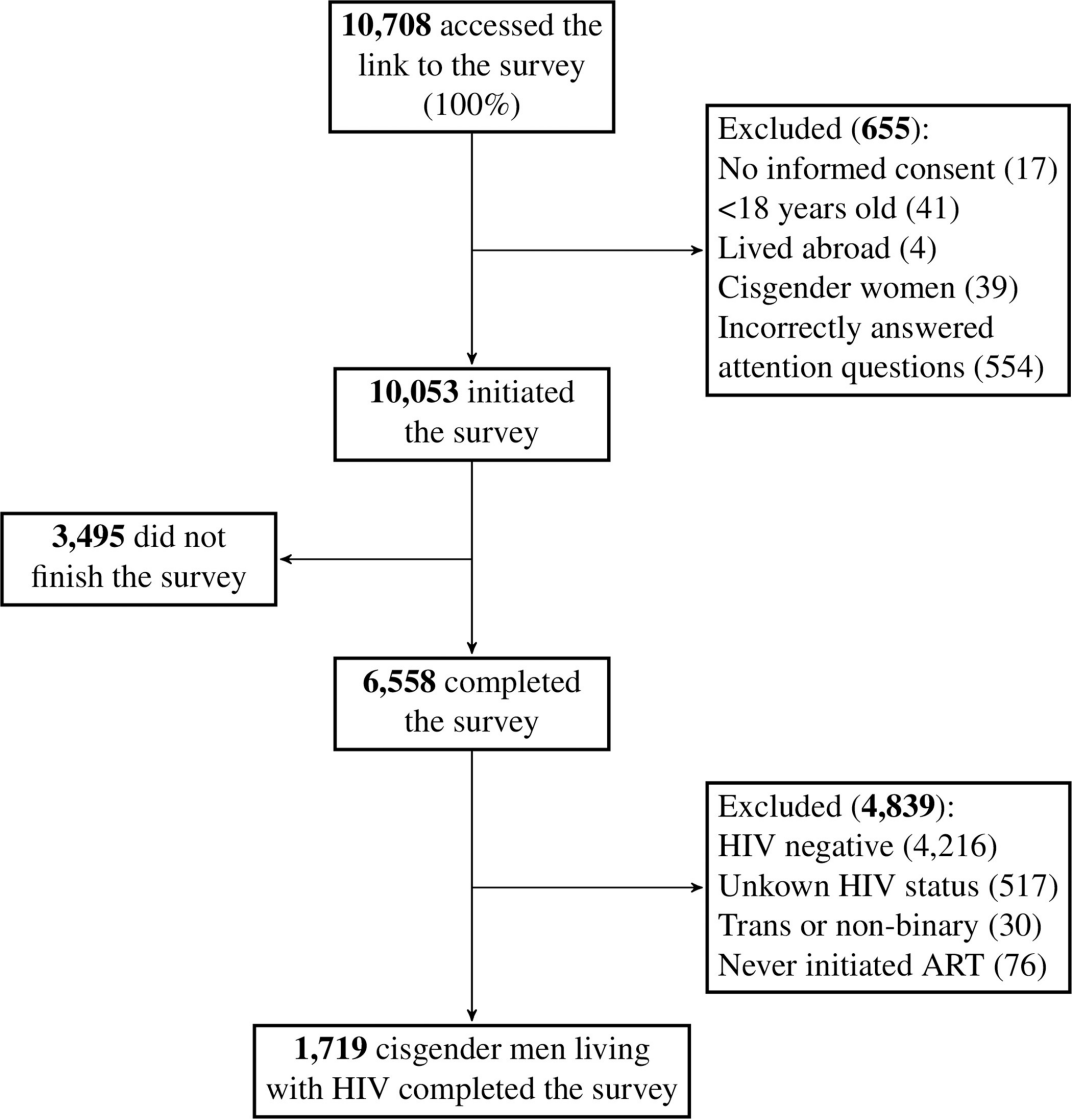
Study population.

In total, 1,719 cisgender MSM reported living with HIV and having initiated ART. Fig 2 depicts the number of participants and all exclusions performed until reaching the study population. Characteristics of the study population are shown in Table 1. Mean age was 38.0 years (SD: 9.9, median: 37, IQR: 30–44), most self-identified as gay (1583, 92.1%), were white (1050, 62.4%), and reported college degree or higher (1178, 69.5%). Almost half of the study population reported moderate household income (784, 45.6%). Mean score on the AUDIT-C scale was 3.7 and 822 (47.8%) participants scored 4 points or more suggesting hazardously drinking. Substance use in the six months prior to participation was more frequent for cannabis (519, 30.2%) and cocaine (299, 17.4%).

**Table 1:** Characteristics of the study population.

	N (%)
Respondents	1719
Age [Mean (SD) Median [IQR]]^a^	37.99 (9.90) 37 [30 – 44]
Age group	
18–24	86 (5.0)
25–29	283 (16.5)
30–39	679 (39.5)
40–49	407 (23.7)
50–59	226 (13.1)
60+	38 (2.2)
Sexual orientation	
Asexual	3 (0.2)
Bisexual/Pansexual	127 (7.4)
Gay/Homosexual	1583 (92.1)
Heterosexual	6 (0.3)
Race	
Asian	21 (1.3)
Black	178 (10.6)
*Pardo* (mixed)	416 (24.7)
Indigenous	17 (1.0)
White	1050 (62.4)
Schooling	
High school or less	519 (30.6)
Undergraduate degree	661 (38.9)
Graduate degree	517 (30.5)
Household income^b^	
Low	461 (26.8)
Middle	784 (45.6)
High	474 (27.6)
AUDIT-C score [Mean (SD) Median [IQR]]	3.65 (2.67) 3 [1 – 5]
AUDIT-C score intervals	
0–3	897 (52.2)
4–12	822 (47.8)
Have you used any of these substances in the past 6 months?	
Cocaine	299 (17.4)
Crack	9 (0.5)
Cannabis	519 (30.2)
Methamphetamine	35 (2.0)
Inhalants	94 (5.5)
Short HIV Stigma scale score	
Personalized HIV stigma [Mean (SD) Median [IQR]]	5.51 (2.53) 5 [3 – 7]
HIV disclosure concerns [Mean (SD) Median [IQR]]	10.69 (1.91) 12 [10 – 12]
Concerns about public attitudes [Mean (SD) Median [IQR]]	9.34 (2.22) 10 [8 – 11]
Negative self-image [Mean (SD) Median [IQR]]	7.29 (2.57) 7 [5 – 9]
WebAd-Q questionnaire on ART adherence	
In the last 7 days, have you taken any of your regimen drugs at times other than those scheduled by your doctor?	
No	1087 (63.2)
Yes	611 (35.5)
I don’t remember	21 (1.2)
In the last 7 days, have you failed to take any of your regimen drugs?	
No	1420 (82.6)
Yes	271 (15.8)
I don’t remember	28 (1.6)
In the last 7 days, have you taken less or more pills of any of your regimen drugs?	
No	1568 (91.2)
Yes	118 (6.9)
I don’t remember	33 (1.9)
Adherence based on WebAd-Q criteria^c^	993 (57.8)
Adherence based on slider	
Score [Mean (SD) Median (IQR)]	96.28 (12.40) 100 [98.5–100]
Marked 100% on virtual slider	1210 (70.4)

^a^SD: standard deviation, IQR: interquartile range.

^b^Household income was based on the ratio of household income relative to the number of minimum wages monthly: low ≤ 2, middle > 2 & ≤ 6, high > 6.

^c^Answered “no” to all three questions.

HIV disclosure concerns and concerns about public attitudes were the HIV-related stigma dimensions most endorsed by participants, with a mean score of 10.69 (SD: 1.91, median: 12, IQR: 10–12) and 9.34 (SD: 2.22, median: 10, IQR: 8–11), respectively. Conversely, personalized HIV stigma was the least endorsed by participants with a mean score of 5.51 (SD: 2.53, median: 5, IQR: 3–7). Negative self-image’s mean score was 7.29 (SD: 2.57, median: 7, IQR: 5–9).

A total of 993 (57.8%) and 1210 (70.4%) participants were considered adherent to ART according to the WebAd-Q scale and the one-item slider question, respectively. In the WebAd-Q scale, not keeping to the scheduled time of ART medications (first item of WebAd-Q) was the item most endorsed by participants (611, 35.5%). In contrast, 271 (15.8%) informed not taking some of their medication and 118 (6.9%) did not comply with the prescribed amount of medication.

Our estimated structural equation model showed good fit (RMSEA = 0.041, 90% confidence interval (90%CI) = 0.039–0.043, CFI = 0.967, TLI = 0.962, SRMR = 0.068), suggesting that the covariance matrix predicted by the model is supported by the underlying data. Construct validity was supported for all latent variables, with especially high standardized factor loadings for the previously validated instruments. Although the loading of race on the socioeconomic status construct was below the 0.5 threshold (0.249), it was kept as an indicator variable due to its relevance in the Brazilian context and to minimize measurement error of its construct (Table 2). Additionally, while the loading for use of crack on the substance use construct was below 0.5 (0.475), it was kept in the model since its removal did not change substantially our fit measures (RMSEA = 0.044, 90%CI = 0.041–0.047, CFI = 0.966, TLI = 0.960, SRMR = 0.062).

**Table 2:** Standardized factor loadings, standard errors (SE) and 95% confidence intervals (95%CI) of the indicators of all latent variables.

	Standardized coefficient (SE)	95%CI
Socioeconomic status		
Household income	0.810 (0.044)	0.724–0.897
Schooling	0.651 (0.037)	0.578–0.724
Race	0.243 (0.036)	0.171–0.314
Alcohol use (AUDIT-C)^a^		
How often do you have a drink containing alcohol?	0.812 (0.012)	0.789–0834
How many standard drinks with alcohol do you have on a typical day?	0.852 (0.011)	0.831–0.873
How often do you have six or more drinks on one occasion?	0.897 (0.009)	0.878–0.915
Substance use		
Cocaine	0.862 (0.036)	0.792–0.931
Crack	0.472 (0.103)	0.269–0.675
Cannabis	0.663 (0.039)	0.586–0.740
Methamphetamine	0.749 (0.053)	0.646–0.853
Inhalants	0.807 (0.043)	0.723–0.892
Short HIV stigma scale		
Personalized HIV stigma		
People I care about stopped calling after learning I have HIV	0.853 (0.011)	0.832–0.875
I have lost friends by telling them I have HIV	0.903 (0.010)	0.884–0.923
Some people avoid touching me once they know I have HIV	0.856 (0.011)	0.834–0.877
HIV disclosure concerns		
I work hard to keep my HIV a secret	0.877 (0.011)	0.856–0.898
Telling someone I have HIV is risky	0.955 (0.009)	0.937–0.972
I am very careful who I tell that I have HIV	0.822 (0.015)	0.792–0.851
Concerns about public attitudes		
Most people believe a person who has HIV is dirty	0.819 (0.014)	0.792–0.846
People with HIV are treated like outcasts	0.757 (0.016)	0.726–0.789
Most people are uncomfortable around someone with HIV	0.832 (0.014)	0.806–0.859
Negative self-image		
I feel guilty because I have HIV	0.689 (0.019)	0.651–0.726
People’s attitude about HIV make me feel worse about myself	0.842 (0.017)	0.808–0.875
I feel I’m not as good a person as others because I have HIV	0.680 (0.023)	0.635–0.724
Adherence to ART		
WebAd-Q questionnaire		
Taken any of your regimen drugs at times other than those scheduled?	0.635 (0.030)	0.576–0.693
Failed to take any of your regimen drugs?	0.993 (0.018)	0.957–1.028
Taken less or more pills of any of your regimen drugs?	0.794 (0.028)	0.740–0.848
Taken 100% of your regimen drugs in the past 30 days?	0.781 (0.024)	0.734–0.829

^a^AUDIT: Alcohol use disorders identification test

**Fig 3:**
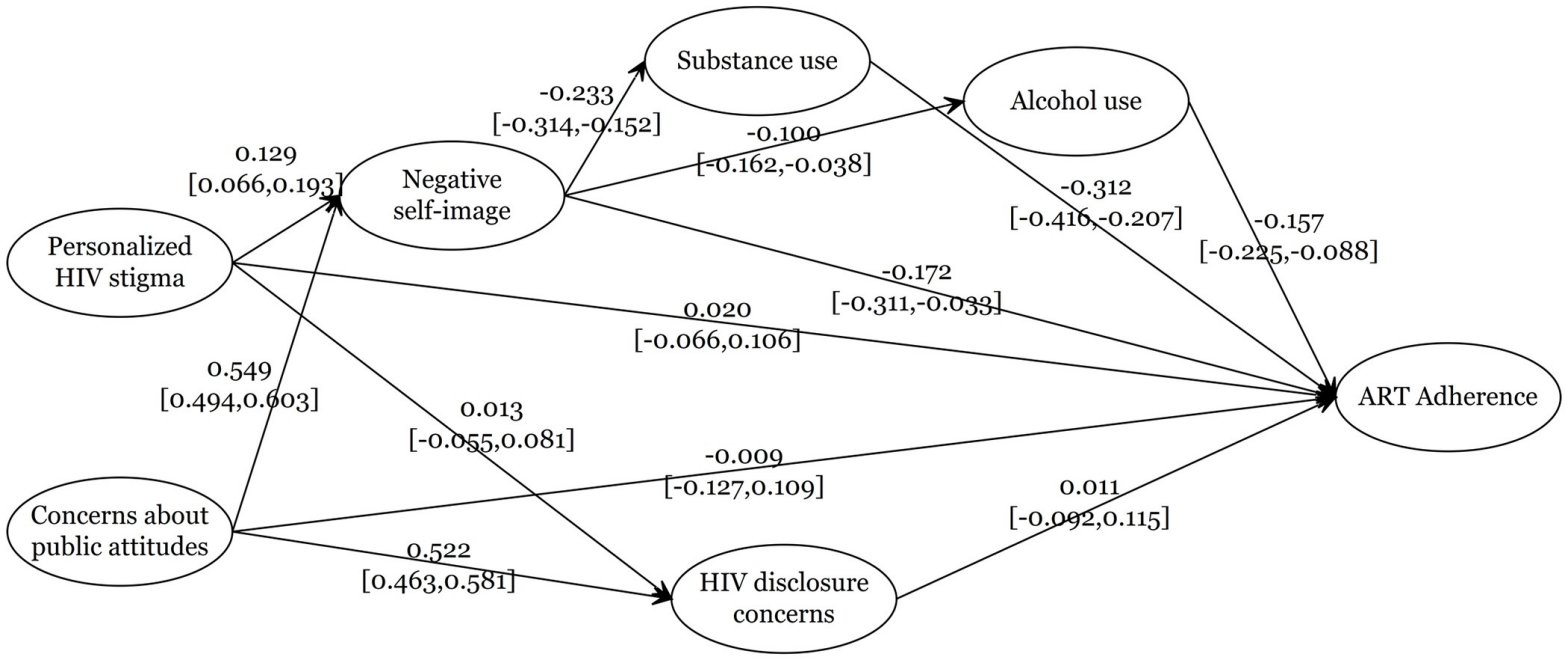
Standardized coefficients (SC) and their 95% confidence intervals (95%CI) of the relationship between HIV-related stigma dimensions and ART adherence. Covariates included in the model were age and socioeconomic status (income, race and schooling).

Fig 3 shows the final model’s standardized coefficients (SC) and their 95% confidence intervals (95%CI) for each pathway. We found that personalized HIV stigma (SC = 0.129, 95%CI = 0.066–0.193) and concerns about public attitudes (SC = 0.549, 95%CI = 0.494–0.603) were both positively associated with negative self-image, which in turn was negatively associated with ART adherence (SC = -0.172, 95%CI = -0.311 - -0.033). In contrast, only concerns about public attitudes (and not personalized HIV stigma) were positively associated with HIV disclosure concerns (SC = 0.522, 95%CI = 0.463–0.581), and the latter was not associated with ART adherence (SC = 0.011, 95%CI = -0.092–0.115), meaning that HIV disclosure concerns were not found to be a mediator between concerns about public attitudes and ART adherence.

Model results also show that negative self-image was negatively associated with substance use (SC = -0.233, 95%CI = -0.314 - -0.152) and alcohol use (SC = -0.100, 95%CI = -0.162 - -0.038). In turn, substance use and alcohol use were both negatively associated with ART adherence, respectively (SC = -0.312, 95%CI = -0.416 - -0.207) and (SC = -0.157, 95%CI = -0.225 - -0.088). These results suggest that negative self-image mediates the association of personalized HIV stigma and concerns about public attitudes with ART adherence through three different pathways (Fig 3 and Table 3).

**Table 3:** Standardized coefficients, standard errors (SE) and 95% confidence intervals (95%CI) for the pathways from personalized HIV stigma and concerns about public attitudes to ART adherence according to the type of effect and the mediators.

	ART Adherence		
	Effect	Standardized coefficient (SE)	95%CI
Personalized HIV stigma			
	Direct	0.020 (0.044)	-0.066–0.106
Through negative self-image	Indirect	-0.022 (0.011)	-0.043- —0.001
Through negative self-image + alcohol use	Indirect	0.002 (0.001)	0.000–0.004
Through negative self-image + substance use	Indirect	0.009 (0.004)	0.003–0.016
Through HIV disclosure concerns	Indirect	< 0.001 (0.001)	-0.001–0.002
Concerns about public attitudes			
	Direct	-0.009 (0.060)	-0.127–0.109
Through negative self-image	Indirect	-0.095 (0.039)	-0.172 - -0.017
Through negative self-image + alcohol use	Indirect	0.009 (0.003)	0.002–0.015
Through negative self-image + substance use	Indirect	0.040 (0.011)	0.018–0.061
Through HIV disclosure concerns	Indirect	0.006 (0.028)	-0.048–0.060

Standardized coefficients and their 95%CIs for all possible associations between our exposure variables (personalized HIV stigma and concerns about public attitudes) and the outcome (ART adherence) according to the type of effect (direct or indirect) and the mediators are shown in Table 3. Neither personalized HIV stigma nor concerns about public attitudes had a direct effect on ART adherence. A significant negative indirect effect for both personalized HIV stigma (SC = -0.022, 95%CI = -0.043 - -0.001) and concerns about public attitudes (SC = -0.095, 95%CI = -0.172 - -0.017) on ART adherence was observed through negative self-image. Examining the serial mediation of negative self-image with alcohol and substance use, all pathways from personalized HIV stigma and concerns about public attitudes showed a significant indirect effect on ART adherence. We found that the personalized HIV stigma was associated with ART adherence through negative self-image and alcohol use (SC = 0.002, 95%CI = 0.000–0.004) and through negative self-image and substance use (SC = 0.009, 95%CI = 0.003–0.016). Similarly, concerns about public attitudes were also associated with ART adherence through negative self-image and alcohol use (SC = 0.009, 95%CI = 0.002–0.015) as well as through negative self-image and substance use (SC = 0.040, 95%CI = 0.018–0.061).

## Discussion

In this cross-sectional study that included 1,719 MSM from Brazil, we explored the potential direct and indirect pathways between multiple HIV-related stigma dimensions and ART adherence. We found that both personalized HIV stigma and concerns about public attitudes were associated with negative self-image which itself was negatively associated with ART adherence. In contrast, only concerns about public attitudes were associated with HIV disclosure concerns, and the latter was not associated with ART adherence. Moreover, we found that negative self-image was associated with alcohol and substance use, both of which, in turn, had a negative effect on ART adherence.

Our results showed that 57.8% to 70.4% of participants were adherent to ART depending on the instrument used to measure it. These findings are similar to prior estimates from Brazil, including a meta-analysis of studies conducted from 2005 and 2016 (64%, 95%CI = 54–73%) [14]. A nationally representative survey of PLHIV conducted in Brazil in 2010 that included 2424 people receiving ART in public HIV-care facilities found lower levels of adherence (39%, 95%CI = 36–42%, this study also used the WebAd-Q instrument) [54]. This difference may result from recent improvements in antiretroviral medications, such as the incorporation of dolutegravir, and from characteristics of our study population, which included predominantly highly educated cisgender MSM. Nonetheless, recent evidence suggests that adherence levels are similar for dolutegravir-based and other regimens [55]. In addition, our results indicated that almost half of the participants (47.8%) scored four or more in the AUDIT-C test, an instrument-based cut-off for hazardous drinking. The Caribbean, Central and South American network for HIV epidemiology (CCASAnet), a large cohort collaboration, found that among 1,274 PLHIV that attended routine clinic visits at a CCASAnet site in Brazil between 2012–2013 17.1% reported consuming 3 or more drinks in the past 7 days [56]. Some studies only report binge drinking, mostly based on the third item of the AUDIT-C test, in which 63.8% of our participants reported drinking six doses or more. A large web-survey conducted in 2019 in Brazil with 11,367 participants found that 68.4% exhibited binge drinking, which was characterized as drinking five drinks or more in a single occasion in the past six months [57]. Cannabis and cocaine use were the substances most participants had used in the previous six months, 30.2% and 17.4% respectively. Similar results were found in the aforementioned web-survey, in which 25.6% and 14.3% of the participants had used cannabis and cocaine respectively in the past six months [57]. However, in the large cohort collaboration also cited above, cannabis and cocaine use were lower, 2.6% and 1.5% respectively [56]. These differences can be due to the same reasons cited above for the prevalence of alcohol use. Taken together, these findings suggest that ART nonadherence and substance use, particularly alcohol use, represent significant challenges in the lives of PLHIV from Brazil.

Our findings also showed how HIV-related stigma burdens our population with participants highly endorsing HIV disclosure concerns, concerns about public attitudes and negative self-image. Similar HIV-related stigma subscales mean scores were found for a sample of 114 Brazilian cisgender MSM users of a geospatial network app (Grindr) in 2019 [46]. Moreover, our findings corroborate previously reported negative associations between HIV-related stigma dimensions and ART adherence [23–25, 27–32]. It also adds to the understanding of the phenomenon by highlighting potential pathways between HIV-related stigma dimensions and ART adherence. Similar to recent research among 203 participants receiving HIV outpatient care in a clinic at the University of Alabama in Birmingham (US) [24], we found that concerns about public attitudes were strongly associated with negative self-image and HIV disclosure concerns suggesting that perceiving one’s community’s devaluation of PLHIV shapes and fuels the fear of experiencing stigma (HIV disclosure concerns) as well as the endorsement that oneself, as a person living with HIV, is of lesser value (negative self-image). It is this negative self-image that then negatively impacts ART adherence, a finding also reported in a study from Zambia that additionally showed that negative self-image negatively impacted adherence motivation, behavioral adherence skills, and self-esteem [27]. Importantly, it may well be that other social and structural factors influence the development of negative self-image [58]. As our study population was composed of cisgender MSM, it is possible that this identity may reinforce negative self-image as this continues to be a discriminated and marginalized population in Brazilian society. A behavioral and biological survey conducted in 2016 in 12 Brazilian capital cities among MSM showed that discrimination due to one’s sexual orientation was highly prevalent, with 64% of the 4,176 participants reporting experiences of discrimination in 13 day-to-day situations [59]. Beyond discrimination, cisgender MSM are subject to high rates of physical and sexual violence, and LGBTQIAPN+ homicides have been steadily increasing in Brazil from 2002 to 2016 [60].

Our serial mediation analysis revealed an unforeseen *negative* effect of negative self-image on substance and alcohol use. It is possible that the endorsement of negative attitudes and beliefs about people living with HIV drives the individual not to socialize much or avoid events or parties, which in turn reduces alcohol and substance use. There is consistent evidence of a negative association between negative self-image and depressive symptoms [23, 25, 27, 28, 29, 50], which in turn is closely related to the individual’s social connectedness [61, 62]. Additionally, there is evidence that participation and connectedness in the LGBTQIAPN+ community is positively associated with alcohol and substance use [63]. Although our participants are older (median age: 37), this could be a plausible explanation for our findings. In a recent systematic review and meta-analysis that evaluated the relationship between alcohol use disorders and HIV-related stigma, no association was observed in the five included studies [64]. To our knowledge, very few studies have analyzed the association between negative self-image and substance use. A study conducted in Georgia (US) with a convenience sample of 358 PLHIV found a positive association between negative self-image and substance use mediated by depressive symptoms [50]. In accordance with prior literature, we also found that both alcohol use and substance use had a strong direct negative effect on ART adherence. A systematic review of the impact of alcohol use on the HIV continuum of care showed that most studies (77% out of the 53 included) found a negative effect of alcohol use on one or more stages of the HIV care continuum [65]. Another study within the large cohort collaboration of PLHIV in Latin America (CCASAnet) found that alcohol use was positively associated with increased loss to follow-up (no contact for at least 1 year before the end of the study–May 1st 2015) [33]. Specifically for ART adherence, a study of 434 PLHIV enrolled in a clinic-based cohort study in Rio de Janeiro between November 2019 and March 2020 found a strong negative direct effect of alcohol use on ART adherence [66]. These findings are also supported by longitudinal studies [34, 67]. Alcohol use is understood to produce negative effects on individual memory, planning, organizational and other cognitive skills, and such impairment may translate into an unintentional ART non-adherence [68]. Moreover, studies have found that people’s beliefs about the toxicity of mixing alcohol and their ART drugs lead them to intentionally not take their ART medication, enhancing the negative effect of alcohol on ART adherence [68, 69]. As for the effect of substance use on ART adherence, some studies reported no association between cannabis use and ART adherence, whereas cocaine, heroin and methamphetamines were negatively associated with adherence [70]. A large cross-sectional study conducted across 12 states in the United States found that alcohol, cocaine, and heroin use were all negatively associated with ART adherence [71]. Additionally, the large cohort collaboration aforementioned found that illicit drug use (marijuana, cocaine, crack or heroin) was negatively associated with ART adherence, and that this association was stronger when alcohol use was also present [56]. Also within the large cohort collaboration, substance use was associated with increased loss to follow-up similarly to the results found for alcohol use [33]. Addressing alcohol and substance use among PLHIV may considerably improve ART adherence. A recent systematic review with meta-analysis found that behavioral interventions targeting multiple HIV barriers have successfully reduced alcohol consumption and risky sexual behavior while also increasing ART adherence [72]. Nevertheless, interventions to address substance use disorders for PLHIV have focused on opioid use disorder with scarce evidence available for the treatment of other substances [69]. Substance use screening in HIV care settings is seen as an efficacious strategy to readily identify substance use disorders and link to treatment [73].

Our findings stress the need of interventions aimed at improving ART adherence levels. Similar to the adherence level exhibited in our sample, Brazilian PLHIV remain distant from the UNAIDS target of 95% adherence [74]. Addressing structural HIV-related stigma is not a simple intervention, still, a recent systematic review of 17 studied interventions aiming to address negative self-image found a statistically significant reduction in HIV-related stigma, which was assessed differently across studies [75]. A narrative review of HIV-related stigma interventions, which included 27 studies, reported 15 successful strategies at reducing one or more HIV-related stigma dimensions [76]. Some interventions were focused on PLHIV themselves, others on partners, children, friends, neighbors or relatives of PLHIV.

Our study has some limitations that need to be stressed. Our sampling strategy was non-probabilistic thus limiting the generalizability of our results. Moreover, study participation required access to a device compatible with geosocial networking (GSN) applications while also requiring a stable internet connection. As such, compared to a broader population of Brazilian MSM, our sample might represent MSM of higher socioeconomic status. Accordingly, our participants were more likely to be residents of the Southeast region of Brazil (the richest and most populous region), highly educated, middle-aged (median age: 37), and to self-identify as White. Nonetheless, nationally representative studies conducted in Brazil in 2023 have shown that smartphone ownership and internet access is widely available across the country: 88% of Brazilians owned a smartphone and 84% had internet access in the past three months [77]. Although the use of race as an indicator of socioeconomic status may be problematic, including in our sample as evidenced by its low loading, in Brazil, it remains an important marker of socioeconomic status, with self-declared Black and Mixed individuals having poorer health indicators, educational outcomes and income [78]. Additionally, the cross-sectional nature of our study implies that we cannot guarantee temporality between exposures and the outcome. To this end, longitudinal evidence has suggested that ART uptake may reduce negative self-image, although mixed results were found for overall HIV-related stigma [79–82]. All of our instruments were self-reported, which could be subject to recall and social desirability bias. Nevertheless, existing evidence points to the reliability of self-reported measures specifically for ART adherence, including for the instrument we used [28, 81]. Additionally, it is worth noting that unmeasured residual confounding is a significant challenge, especially in observational, cross-sectional, self-reported studies such as the present one. We have attempted to account for some of these factors including socioeconomic status and age. Lastly, depression or depressive symptoms have been shown to mediate the relationship between HIV-related stigma (mostly negative self-image) and ART adherence [23, 28]. However, we did not collect information about depression symptoms in this study.

Despite these limitations, this study has several strengths. To our knowledge, this is the first study to investigate the association between HIV-related stigma and ART adherence in Brazil. Our results shed light on the relationship between HIV-related stigma and ART adherence in a middle-income country, and may help tailor interventions focused on the Brazilian reality. Furthermore, we employed a multidimensional measure of HIV-related stigma and used a structural equation model. This approach allowed our analysis to enhance the comprehension of the interplay between the various dimensions of HIV stigma. Lastly, it is noteworthy that both HIV-related stigma dimensions and ART adherence were assessed by previously validated instruments.

## Conclusions

Our research underscores the critical need for multifaceted interventions to combat HIV-related stigma at both individual and societal levels. At the individual level, there are multiple psychological approaches that may prove useful in decreasing negative self-image such as cognitive-behavioral psychotherapy, motivational interviewing, and acceptance and commitment therapy. Motivational interviewing, client-health provider psychotherapeutic collaborative approach that focuses on exploring ambivalence and building motivation for change, could be especially useful for understanding internalized stigma and the potential benefits of self-acceptance. Acceptance and commitment therapy, on the other hand, could help PLHIV by increasing acceptance to a life with HIV while committing to living a fulfilling life aligned with their values. Moving beyond individual interventions, public policy efforts offer a broader approach to dismantling structural stigma. Awareness campaigns through various media channels, integration of anti-stigma (HIV-related, and gender and sexual diversity) curriculum into schools, and training for professionals, especially healthcare providers, social workers and law enforcement agents compose pragmatic actions for tackling structural stigmatization.
